# It's time to re-evaluate the reporting of common measures from isokinetic dynamometers: isokinetic for torque, isotonic for power

**DOI:** 10.3389/fspor.2025.1472712

**Published:** 2025-02-04

**Authors:** Brennan J. Thompson

**Affiliations:** ^1^ Neuromuscular Research Laboratory, Kinesiology and Health Science Department, Utah State University, Logan, UT, United States; ^2^ Movement Research Clinic, Sorenson Legacy Foundation Center for Clinical Excellence, Utah State University, Logan, UT, United States

**Keywords:** strength testing, muscle function, biodex, muscle assessment, peak torque, mean torque

## Abstract

Isokinetic dynamometry is commonly used to provide an objective and reliable assessment of muscle function across a variety of clinical, athletic and research settings. Important muscle function variables that are commonly assessed are torque- and power- related measures. Isokinetic mode is overwhelmingly used to provide these variables, and has been so for decades; however, this mode may not be the best suited to examine power variables. The article aims to explore this issue through conceptual evaluation and empirical results using unpublished data. The implication is that due to the almost complete lack of unique information that power provides additional to torque in isokinetic mode, the isotonic mode is better suited to assess power for functional, operational, and methodological reasons. Thus, muscle function tests on an isokinetic dynamometer provide more fitting and useful data when isokinetic mode is used to determine torque measures, and isotonic mode is used to provide power measures.

## Introduction

**Figure 1:**
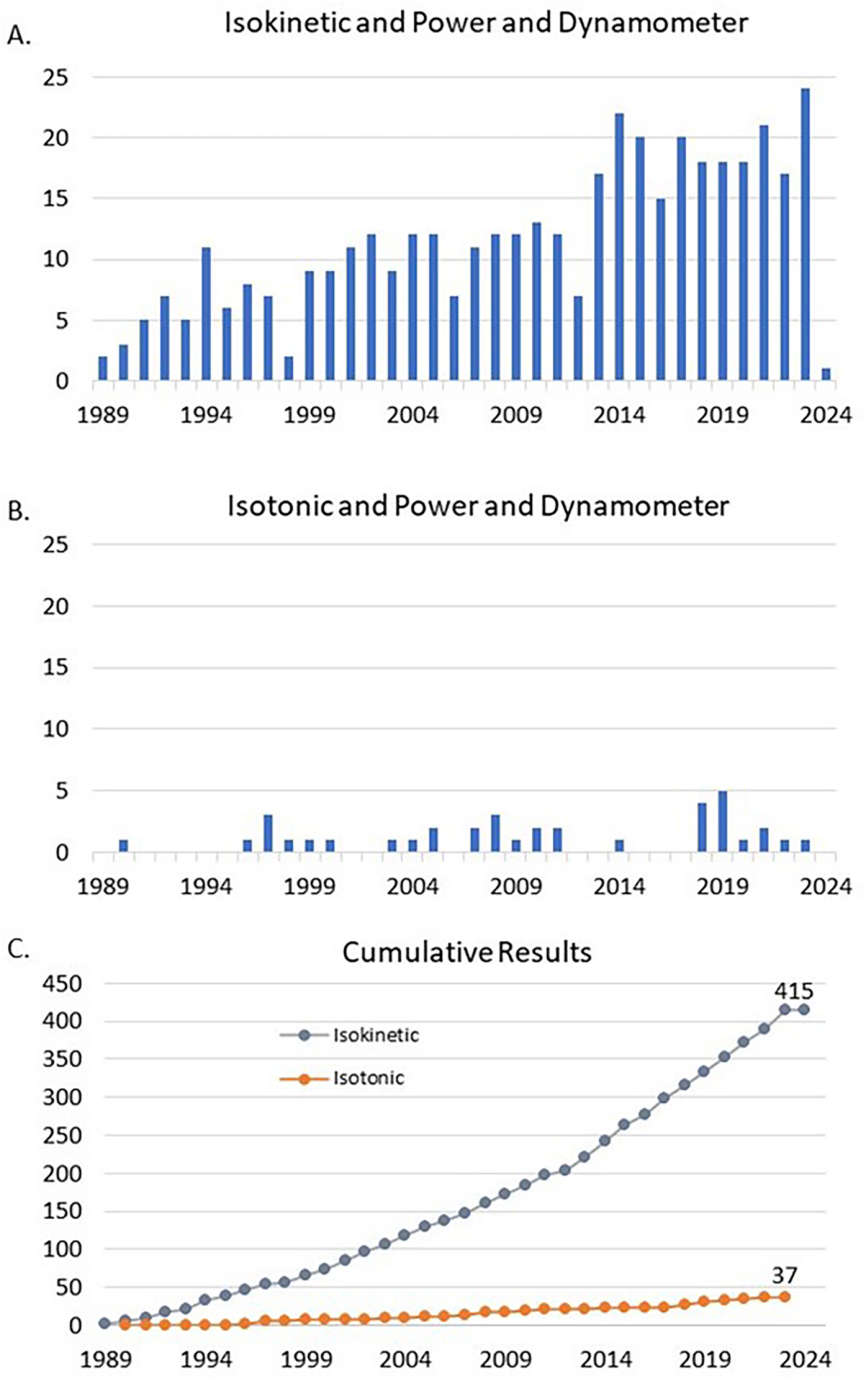
Presents the annualized search results from the Pubmed database for the search terms, **(A)** “*Isokinetic and Power and Dynamometer”* and **(B)** *“Isotonic and Power and Dynamometer”.* Panel **C** presents the cumulative search results for these terms to February of 2024.

Isokinetic dynamometry is commonly used as a muscle function assessment tool across a multitude of clinical, athletic, and research settings. It is generally considered as the gold standard for muscle function assessment due to its objective accuracy, reliability, and standardized controls (1–3). Other advantages of this form of muscle function testing include the capability of providing a multitude of parameters from even just a single repetition, such as peak torque (PT), mean torque, work, peak power (PP), mean power (MP), angle at PT, etc. Because of these features it is by a wide margin the most commonly employed test mode that is used when testing on isokinetic-capable dynamometers. To illustrate the mode reporting discrepancy, Figure 1 shows Pubmed search results comparing “isokinetic” vs. “isotonic” dynamometer power variable terminology, where isokinetic returned more than 10-fold the number of results (there were only 37 results for the “isotonic” dynamometer power phrase, since 1989).

Isokinetic dynamometers offer versatility beyond traditional isokinetic assessments, enabling various modes of muscle function evaluation including isometric, eccentric-only, and isotonic muscle actions. In isotonic mode, the dynamometer applies a “constant resistance,” which is similar in resemblance to the loads encountered in traditional strength tests with free weights or machines. This mode more accurately (vs. isokinetic) reflects the load dynamics commonly experienced in daily activities, which typically involve both concentric and eccentric muscle actions in an environment where movement velocity is not constrained (3). Thus, the main difference between isokinetic and isotonic testing modes are that the former involves a constant velocity but variable resistance, whereas the latter is a constant load and variable velocity. The differences between these two concentric dynamometer-based test modes and how they interact with the most common output measures, namely torque and power, are important; yet the implications of these differences has received relatively little attention in the context of isokinetic dynamometry assessments and the associated reported outcomes. Therefore, the purpose of this brief report is to employ conceptual principles and evidence-based data to highlight and compare the advantages and disadvantages of these two modes in regards to the torque and power output measures they each provide, and to describe how they may complement each other when integrated to provide more comprehensive evaluative muscle function assessment information.

## Isokinetic vs. isotonic operational principles and physiological implications

Because of the dynamometer-imposed velocity control that characterizes the isokinetic mode, the velocity of the limb is of course constrained precisely to the predetermined velocity limit throughout a portion of the range of motion, with the portion of the set constant velocity being variable, depending on the velocity (i.e., isokinetic range excluding acceleration and deceleration phases, which are longer or shorter for faster or slower velocities). This constraining of the velocity is an important, and perhaps too often overlooked factor in regards to the effect that it has on the velocity dependent outcome measures, which primarily includes the commonly reported power measures from isokinetic mode tests. Indeed over 30 years ago observant investigators (4–6) well described how ultimately PT and PP were nearly perfectly associated when they were both derived from isokinetic assessments. This fact prompted one investigator (4) to suggest that measures of power may be “superfluous” when combined with PT, suggesting that practically no new meaningful information was provided by adding the second parameter (power).

Despite those early useful observations, it is rather remarkable that across the many decades and with the prolific research output involving these isokinetic-related measures, that the consideration of how this velocity constraining effect may impact the output variables has not been given as much consideration as perhaps it should. In fact, power is still a commonly reported outcome from isokinetic tests. The reasons for this are likely largely due to dynamometer research reporting conventions, along with the convenience of the software outputs. It is time to revisit the issue raised by early investigators and advocate for a more comprehensive approach to reporting key muscle function outcomes from dynamometer testing protocols. By adopting a more applicable and robust reporting framework, we can enhance the utility and relevance of these measures, maximizing the dynamometer's potential as a powerful tool for evaluating muscle function.

## Power and torque parameters in isokinetic and isotonic contexts

**Figure 2:**
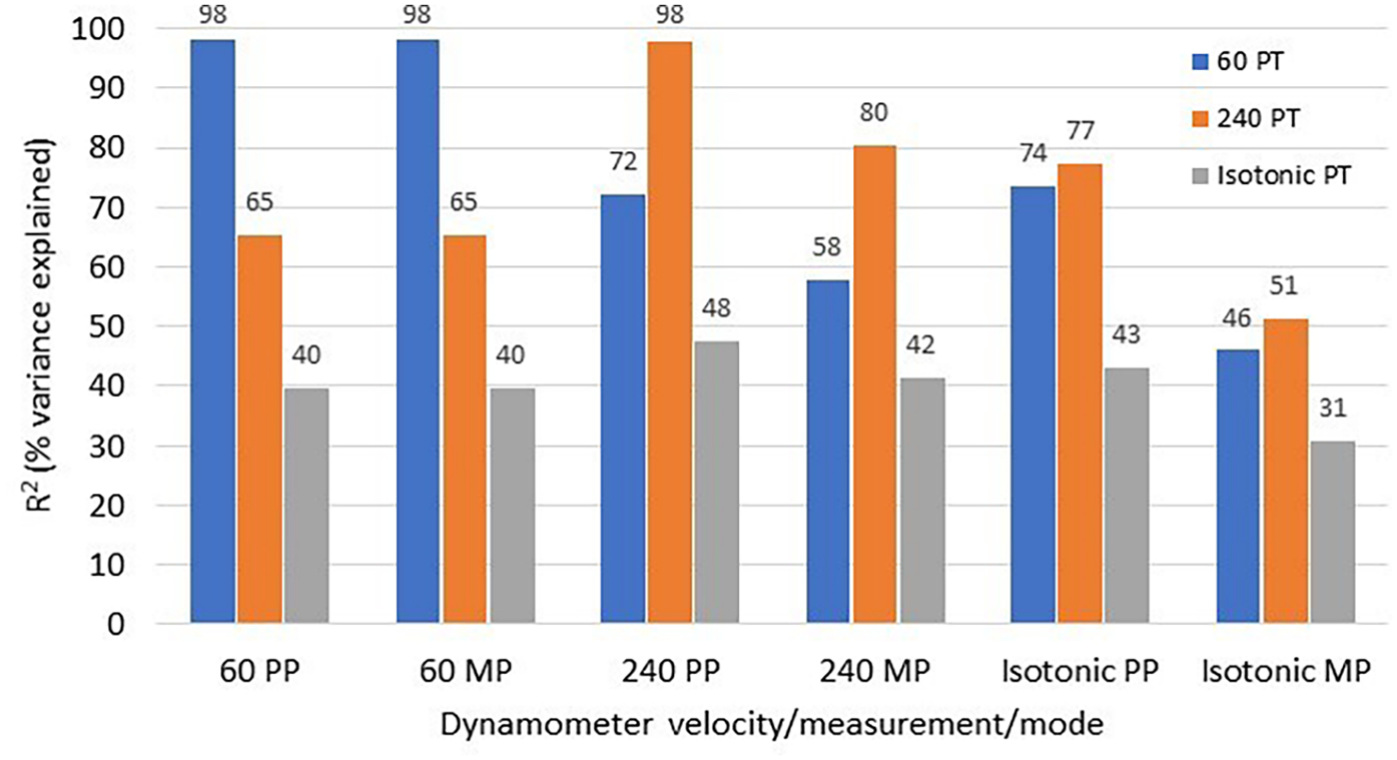
Presents the coefficient of determination (*r*^2^) values for the relationships between the peak torque (PT), peak power (PP) and mean power (MP) dynamometer output measures across separate isokinetic tests at 60 and 240 deg/s and isotonic muscle action modes. Data is from 52 adult participants that performed all muscle contraction modes, in random order, with a 2-min rest period between conditions, and following a familiarization session on a Biodex dynamometer (unpublished data). The coefficients are represented in three series (blue is 60 deg/s PT, orange is 240 deg/s PT and gray is isotonic PT set at 25% of maximum isometric strength) and these series data show the relationships that correspond to the respective *x*-axis titled variable. Note how the PT isokinetic measures nearly perfectly explain the variance in their respective isokinetic PP measures, and how these relationships are markedly diminished when examining them for all of the isotonic mode variables. All correlations were statistically significant at the *P* < 0.001 level.

The principle underlying the relationship between torque and velocity on power becomes disadvantageous in an isokinetic context, at least in terms of where the usefulness of power in a human performance domain is concerned. Because power is the product of force and velocity, constraining velocity to a set limit would then leave torque as the only factor that the resultant power is contingent upon. Put another way, if velocity is set the same for every contraction and/or across all participants, then the only factor that is varying power, is varying the torque output. This is the reason for why PT and power in an isokinetic movement are essentially almost perfectly related (Figure 2). This is problematic when it comes to muscle performance testing because, at best it wastes resources on unnecessary analyses and creates unnecessary “bulk” in numerical test reports (4), and at worst it may provide a false sense of what the true power capabilities really are, thereby providing an erroneous profile of one's true muscle function status. The latter is a counterproductive consequence to the point of the test in the first place, due to its misleading potential, and it is what particularly needs to be avoided.

It may be argued that if PT and power move in near-perfect alignment during isokinetic tests, reporting PT alone could suffice. This rationale, presented in earlier studies, was considered a practical and non-redundant approach to reporting data derived from isokinetic assessments (4). However, this approach raises a critical limitation when aiming for a comprehensive evaluation of muscle performance.

Power is a highly significant attribute in sports, daily functional activities, and health-related outcomes (7–10). In fact, extensive evidence suggests that power may surpass strength in its relevance to athletic performance, functional status, and the detection of clinical impairments (3, 7, 9, 10). Simply disregarding power in reporting, on the basis that it overlaps with PT during isokinetic tests, would undermine its unmatched utility. Such a practice fails to leverage the unique and essential information that power can provide.

To illustrate this point, data (unpublished) were analyzed from 52 adult male and female participants who performed isokinetic tests at 60°/s and 240°/s, as well as an isotonic test at 25% of maximum isometric strength. Figure 2 presents the coefficients of determination (*r*^2^) for the relationships among PT, PP, and MP across different velocities and testing modes. The data demonstrate the near-complete variance explained by PT on PP for isokinetic conditions, but this relationship is markedly reduced in the isotonic mode. This indicates that while PT and PP offer no unique information during isokinetic tests, isotonic power measures provide distinct data and variance from PT. This distinction is of course highly desirable, as it highlights the value of isotonic power measures in muscle function assessments, providing critical insights that are not captured by isokinetic PT alone.

Power is unquestionably a critical and worthwhile metric for evaluating one's muscle performance. To maximize its functional relevance, power should be assessed in a velocity-unconstrained manner. Indeed, in addition to its functional relevance, assessing power is desirable precisely because it does not represent the exact same characteristics as PT as per its unique sensitivity to a number of health, sport performance, and functional aspects (as described above). The issue, therefore, lies in determining how to measure power using an isokinetic dynamometer without allowing it to be limited or overly influenced by its correlation with PT.

The isotonic mode of dynamometry provides a convenient solution by better decoupling torque and power measurements, allowing each metric to best occupy its specific domain in accordance to its likely dynamometer-based assessment purpose (e.g., isokinetic for standardizing velocity-specific strength, and power for its functional implications).

Although it should be noted that there may be cases where reporting both PT and power from isokinetic assessments is warranted—such as when the need arises to directly compare with studies that have exclusively reported the power measure with isokinetic mode. For instance, the two measures are expressed in different units (Nm for PT and Watts for power), making direct comparisons with studies reporting only power values challenging. Nevertheless, this paper contends that the most effective way to utilize power as a complementary measure is through isotonic mode assessments. Even when isokinetic power is reported, including isotonic power alongside it ensures that power retains its unique functional utility, avoiding redundancy with PT and enhancing the depth and applicability of muscle function evaluations.

That said, isotonic mode on an isokinetic dynamometer is not without limitations. In particular, PT as measured in isotonic mode may not provide highly meaningful information for certain comparative purposes. This limitation arises because isotonic PT values are constrained by the constant external load; in other words, PT mostly reflects the fixed external load of the system and does not vary significantly within a constant load system when using a submaximal load. Instead, it is the velocity that varies with changes in applied force.

An illustrative example comes from the dataset discussed earlier, where young and older participants demonstrated a 28% difference in isometric strength, as expected. Yet, their isotonic PT values were nearly identical (64.2 vs. 63.6 Nm), highlighting the limitation of isotonic PT for discriminating between groups with significant strength differences. Consequently, while isotonic mode is well-suited for assessing power and velocity, it appears less useful for evaluating PT as a discriminating variable when assessed at relatively low submaximal loads.

The influence of higher submaximal relative isotonic loads on the utility and discriminative ability of PT remains poorly understood. Future studies should investigate whether PT serves as a more effective discriminative measure when evaluated under these less-explored isotonic dynamometer conditions. Additionally, more studies are required to investigate the practical aspects of how best to incorporate an isotonic muscle assessment protocol alongside an isokinetic protocol. Key factors such as specific methods (sets, reps, rest periods), reliability factors, and fatigue effects should be thoroughly explored to develop an optimized and time-effective protocol that maximizes data usefulness, accuracy, and practicality.

## Discussion

The solution to this mode by measure dilemma is to use both isokinetic and isotonic modes when aiming to comprehensively describe muscle function capabilities. Rather than report all metrics solely from the isokinetic mode, it would be more prudent to use isokinetic for the advantages it provides in giving a suitable PT measure (i.e., to independently and reliably provide PT capacities without the confounding influence of velocity), but incorporate isotonic contractions for the express purpose of providing the power measures. Such a protocol would yield more fruitful and relevant comprehensive muscle performance data sets whereby both PT and power would be suited to their distinct purposes, and each would give some unique information on muscle function. The combination of these modes is therefore an optimal way to capture the best of each, and would not require more than a few extra sets of testing (requiring < 5 min of time to add 2–3 isotonic sets to the isokinetic testing). Hence, isokinetic mode should be used primarily for PT measures and isotonic for power.

## Data Availability

The data analyzed in this study is subject to the following licenses/restrictions: just reported as aggregate values. Requests to access these datasets should be directed to brennan.thompson@usu.edu.
